# An assessment of the use of Hepatitis B Virus core protein virus-like particles to display heterologous antigens from *Neisseria meningitidis*

**DOI:** 10.1016/j.vaccine.2020.03.001

**Published:** 2020-04-03

**Authors:** Sebastian Aston-Deaville, Emil Carlsson, Muhammad Saleem, Angela Thistlethwaite, Hannah Chan, Sunil Maharjan, Alessandra Facchetti, Ian M. Feavers, C. Alistair Siebert, Richard F. Collins, Alan Roseman, Jeremy P. Derrick

**Affiliations:** a Lydia Becker Institute of Immunology and Inflammation, School of Biological Sciences, Faculty of Biology, Medicine and Health, Manchester Academic Health Science Centre, University of Manchester, Manchester M13 9PL, UK; b National Institute for Biological Standards and Control, Blanche Lane, South Mimms, Hertfordshire EN6 3QG, UK; c Electron Bio-Imaging Centre, Diamond Light Source, Harwell Science & Innovation Campus, Didcot, Oxfordshire, UK

**Keywords:** Neisseria meningitidis, Virus-like particle, Electron microscopy, Structural vaccinology

## Abstract

*Neisseria meningitidis* is the causative agent of meningococcal meningitis and sepsis and remains a significant public health problem in many countries. Efforts to develop a comprehensive vaccine against serogroup B meningococci have focused on the use of surface-exposed outer membrane proteins. Here we report the use of virus-like particles derived from the core protein of Hepatitis B Virus, HBc, to incorporate antigen domains derived from Factor H binding protein (FHbp) and the adhesin NadA. The extracellular domain of NadA was inserted into the major immunodominant region of HBc, and the C-terminal domain of FHbp at the C-terminus (CFHbp), creating a single polypeptide chain 3.7-fold larger than native HBc. Remarkably, cryoelectron microscopy revealed that the construct formed assemblies that were able to incorporate both antigens with minimal structural changes to native HBc. Electron density was weak for NadA and absent for CFHbp, partly attributable to domain flexibility. Following immunization of mice, three HBc fusions (CFHbp or NadA alone, NadA + CFHbp) were able to induce production of IgG1, IgG2a and IgG2b antibodies reactive against their respective antigens at dilutions in excess of 1:18,000. However, only HBc fusions containing NadA elicited the production of antibodies with serum bactericidal activity. It is hypothesized that this improved immune response is attributable to the adoption of a more native-like folding of crucial conformational epitopes of NadA within the chimeric VLP. This work demonstrates that HBc can incorporate insertions of large antigen domains but that maintenance of their three-dimensional structure is likely to be critical in obtaining a protective response.

## 1 Introduction

The Gram-negative bacterium *Neisseria meningitidis* is one of the leading causes of bacterial meningitis in infants, in which the incidence of patient mortality (10%) or the development of sequelae (50%) is tragically high [1]. Strains are classified according to serogroup, based on capsular polysaccharide, with groups A, B, C, W, X and Y being the major circulating strains worldwide [2], [3], [4]. Polysaccharide (conjugate) vaccines against all these groups, with the exception of group B, have been or are being developed. The serogroup B polysaccharide has not been included in this approach due to its similarity with human foetal neuronal antigens that made it poorly immunogenic [5]. Initial attempts to develop a vaccine in response to region-specific outbreaks of capsular group B *N. meningitidis* utilised outer membrane vesicles (OMVs) [6], [7]. However, outer membrane protein Porin A (PorA), which is typically the dominant component against which an immune response is generated, is a highly variable antigen with little to no cross-reaction between variants [8]. This problem led to a renewed search for surface-exposed protein antigens by Reverse Vaccinology, an approach which combined *in silico* analysis of genomic sequences with the testing of recombinant protein antigens to identify optimal vaccine candidates [9], [10]. The 4CMenB (Bexsero) vaccine, which was introduced in the UK routine childhood immunisation schedule in 2015, incorporates three such recombinant antigens in addition to an OMV component: Factor H binding protein (FHbp), *Neisseria* adhesin A (NadA), and *Neisseria* heparin-binding antigen (NHBA) [11]. Other methods, such as antibody subtraction, simultaneously identified FHbp, confirming its potential as a vaccine candidate, and were used as the basis for the independent development of the bivalent FHbp vaccine rLP2086 (Trumenba) [12]. This vaccine, however, is not currently licensed for use in the infant population.

FHbp is a lipoprotein that binds serum factor H, as an immune evasion mechanism, through a protein surface which mimics carbohydrate ligands [13]. There is a diverse population of fHbp sequences that are, by convention, broadly divided into two or three variant groups based on their sequence relatedness, although further genetic diversity has been documented within these groups. Variants within group 1, including the variant incorporated in the 4CMenB vaccine, demonstrate cross-reactivity with antibodies generated against variants within the same group but not against those of groups 2 and 3 [14]. For this reason, the Trumenba vaccine incorporates two variants, one each from subfamilies A and B [15]. The structure consists of two domains: a C-terminal 8-stranded β-barrel and a 6-stranded antiparallel β-sheet packed against a short coiled coil at the N-terminus [16]. NHBA shares a similar β-barrel fold to the FHbp C-terminal domain, suggesting that they may be related in origin [17]. NadA is a member of the widespread bacterial autotransporter family and adopts a trimeric structure, dominated by an extensive region of coiled coil, with a small α/β domain at the tip of the structure which is distal from the outer membrane surface [18], [19]. All three antigens can induce the production of bactericidal antibodies following immunization [11], [20]. However, the genetic diversity often associated with sub-capsular antigens has implications for vaccine coverage where the genetic epidemiology of disease isolates changes frequently, both temporally and geographically. The ease of reformulation of vaccines based on sub-capsular antigens therefore needs to be considered.

Virus-like particles (VLPs) are well established, flexible protein platforms for the display of vaccine antigens, on account of their large, repetitive assembly, which gives them inherent stability and useful immunogenic properties (eg adjuvant-like effects) [21], [22], [23]. The use of VLPs for vaccination against their cognate viral diseases is well established, with hepatitis B virus and human papillomavirus being the most notable examples [24], [25]. VLPs have also been widely employed as a platform for the display of heterologous peptide antigens (derived from bacterial, eukaryotic, and even unrelated viral pathogens) that can be expressed in recombinant form [26]. In this way, the viral assembly enhances immunogenic responses against the antigen insert through an adjuvant effect [27].

Hepatitis B core antigen (HBc) is one of the best established VLPs, and has been confirmed to be a safe platform that has seen use in both animal models and at least two clinical trials [28]. The capsid protein is small (ca. 150 residues) and its structure is well established by crystallography and cryoelectron microscopy [29], [30], [31]. Heterologous antigens can be inserted into the major immunodominant region (MIR), which is located at the tip of surface α-helical spikes, or the C-terminus, whilst retaining the ability to assemble into a stable particle. Some of the more prominent modifications include: the removal of nucleic acid binding capability, reduced reactogenicity against the intrinsic HBc T-cell epitopes, and enhanced stability of the capsid structure [32], [33]. We reasoned that introduction of *N. meningitidis* antigens into a VLP may confer specific advantages in providing protection against meningococcal disease that are not found when the antigen is expressed alone.

We therefore set out to examine the feasibility of incorporation of two well-established meningococcal vaccine antigens into a single VLP. We show, somewhat surprisingly, that it is possible to engineer two different and substantial antigen domains into a single HBc polypeptide chain and retain competent assembly of the capsid. Mouse immunization experiments show that antibodies are raised against both antigens, but only one is effective in eliciting a bactericidal antibody response. We propose that this observation correlates with the folded state of the antigen and discuss the general implications of this proposition for VLP engineering more generally.

## 2 Results

### 2.1 Expression of HBc-based VLP constructs

**Fig. 1:**
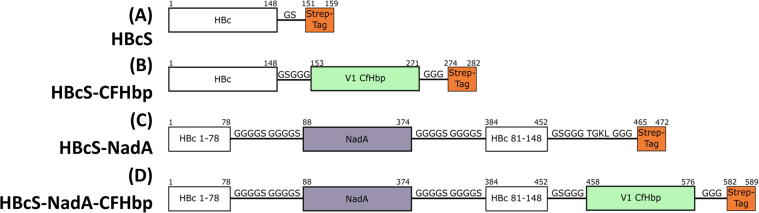
VLP-antigen constructs. Constructs contain linker regions composed of glycine and serine residues to provide flexibility and a Strep-tag for purification. CFHbp sequence refers to residues H157 to Q274 of the FHbp ORF from strain MC58 (PubMLST allele designation: 1); this corresponds to the C-terminal domain comprising approximately half the mature protein [13]. NadA sequence refers to residues A26 to G309 of the NadA ORF from strain MC58 (PubMLST allele designation: 1); this corresponds to the N-terminal globular and subsequent coiled-coil domains, encompassing all of the soluble parts of the mature protein [18].

A range of VLP and chimeric VLP-antigen constructs were designed, based on the core structure of a C-terminal truncated HBc peptide, by omitting the disordered RNA-binding domain which has been previously demonstrated to improve expression [34]. A Strep-tag was inserted at the C-terminus for ease of purification, generating the construct HBcS (Fig. 1). The antigen-coding sequences were incorporated into HBc VLP either at the C-terminus or the MIR. The choice of antigen (and variant) was informed by the successful use of three soluble recombinant antigens in the 4CMenB vaccine formulation; constructs were designed with variations in antigen length and insertion site using combinations of variant 1 FHbp, NadA and NHBA. Linker sequences were designed to flank the inserted antigen sequence in order to impart some degree of flexibility into the structure, to allow the VLP to accommodate the insertion without compromising assembly. The VLP fusion constructs were trialled for their ability to express soluble protein (Table S1). It was found, however, that many were not able to generate assembled VLPs; constructs incorporating NHBA were particularly resistant. We noted that the domain structure of the inserted antigen and insertion point were important factors in the isolation of assembled VLPs. For example, incorporation of the full length or N-terminal domain of variant 1 FHbp at the HBc C-terminus removed the ability of the construct to form assembled VLPs. Insertion of the C-terminal domain of variant 1- but not variant 3- FHbp was successfully incorporated, however. Somewhat surprisingly given its trimeric nature, NadA was stably accommodated at either the MIR or C-terminus of HBc. This observation led us to fabricate a construct incorporating both antigens (HBcS-NadA-CFHbp), which also formed intact VLPs (Fig. 1 and Table S1).

**Fig. 2:**
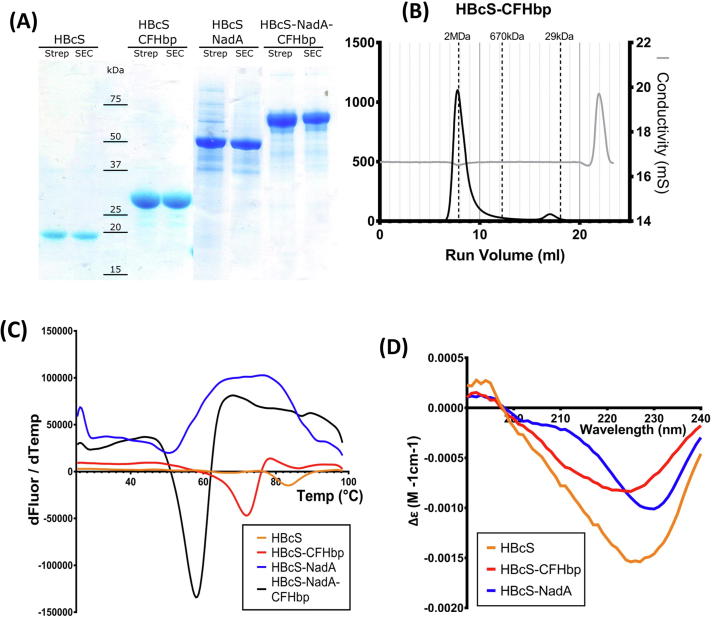
Characterization of HBcS-antigen fusions. (A) SDS-PAGE of purified HBcS-antigen fusions. (B) Exemplar size exclusion elution chromatogram from the purification of HBcS-CFHbp. Chromatography was performed in 1 × PBS using the Superose6 (GE Healthcare). Vertical dashed lines indicate the elution volume of mass standards. (C) Thermofluor assay of purified HBc-antigen fusions. Data are presented as a plot of increasing assay temperature (°C) against the mean derivative of fluorescence (AU) with respect to temperature (dF/dT). Data are the means of three repeats. (D) Circular dichroism spectra for each HBcS-antigen fusion.

All four constructs detailed in Fig. 1 were purified in good yield (approximately 8 mg protein per litre of cultured cells; Fig. 2A). Size exclusion chromatography provided evidence that all four constructs assembled into VLPs (Fig. 2B). Assembled VLPs would be expected to elute in the void volume whilst lower molecular weight assembly intermediates, of which HBc dimers are expected to be the major component [35], elute later. The impact of antigen insertion on VLP stability was also examined, using an assay which records a discontinuity in fluorescence of an extrinsic probe as a function of temperature [36] (Fig. 2C). Although the incorporation of antigen did reduce the apparent melting point (T_m_) of the VLP constructs trialled, the measured melting points were still in excess of 45 °C: 83 °C (HBcS), 72 °C (HBcS-CFHbp), 50 °C (HBcS-NadA) and 58 °C (HBcS-NadA-CFHbp). Storage at −80 °C did not have an effect on T_m_ values.

### 2.2 Cryoelectron microscopy structure determination of HBcS-NadA-CFHbp

The simultaneous incorporation of NadA and the C-terminal domain of FHbp into HBcS generated a modified viral protein which is 3.7 times larger than the native scaffold (Fig. 1). Although the stability of the assembly is compromised to some extent (Fig. 2C), our initial EM observations established that the VLP assembly still forms. Accommodation of the NadA trimeric protein antigen ostensibly poses challenges for HBc assembly, as it potentially introduces an additional 3-fold symmetry axis into the assembly.

**Fig. 3:**
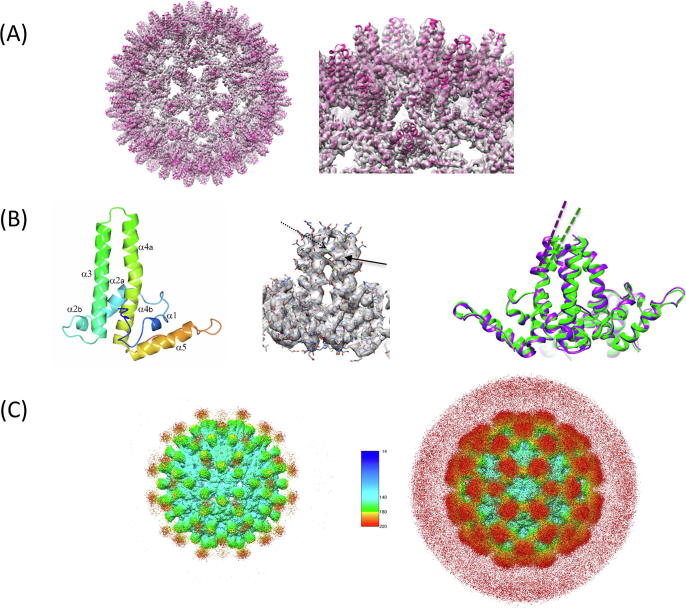
Cryoelectron microscopy of HBcS-NadA-CFHbp. (A) 3D reconstruction showing the higher density core structure. The crystal structure of the T = 4 HBV core protein from Wynne *et al.* is overlaid [30]. The helical nature of the spike structure is clearly evident directly in the EM map. The wild type core structure is strongly preserved in the new construct, though some deviation from the native crystal structure at the tips of the spikes can be seen. The tip to tip diameter of the VLP core is ~35 nm. The right panel shows an enlarged view with detail of the dimeric spike structure. The threshold for the map is 2 (8.6σ above mean). (B) Left panel: ribbon plot of the core shell protein monomer, with the different helical segments labelled (Protein Data Bank file 1QGT), with a blue-red color gradient from the N- to C-terminus. Middle panel: view of a dimeric spike, showing the refined atomic model superimposed on density. The broken and solid arrows indicate two important protein side chains involved in stabilising the dimer through aromatic ring stacking (Trp71 and Tyr88 respectively). The threshold for the map is 2 (8.6σ above mean). Right panel: Ribbon representation, of a different view of the dimeric spike, showing the displacement and slight change in the orientation of the axis (dashed lines) of one of the helixes forming the spike (helix 4a). The remodeled new structure is in purple, while the original crystal structure is shown in green. (C) Left: density map, coloured by radius, contoured at threshold value of 0.5 (2.1σ above mean) to illustrate density originating from the NadA component. Density attributable to NadA is shown in red, and falls on the 3-fold symmetry axes. Right: the same map contoured at 0.33 (1.4σ above mean), to highlight the NadA density, and therefore with a higher background. The key shows the radius in Å. (For interpretation of the references to color in this figure legend, the reader is referred to the web version of this article.)

We were therefore interested to know whether incorporation of dual antigen domains and the corresponding increase in overall mass compromised VLP assembly. Consequently, HBcS-NadA-CFHbp was subjected to higher resolution structure determination by cryoelectron microscopy (Fig. 3A & B and Fig. S1-3). Processing of single particle data showed that the T = 4 form of the capsid predominated. Examination of the cryoelectron map shows a clearly resolved HBV icosahedral core shell structure, with additional density clouds present on the 3-fold and pseudo 3-fold axes representing the inserted antigen (Fig. 3C). Although the NadA insert density is weaker and appears disordered, it is not necessarily unfolded: CD data were consistent with a high proportion of α-helix (Fig. 2D). NadA constitutes nearly 50% of the mass of HBcS-NadA-CfHbp and the native NadA trimer is formed from a trimeric coiled coil [18], [19]. The density at the strict 3-fold axes is weaker than the *pseudo* 3-fold positions: this indicates that the NadA domains may retain their trimeric state, and that the flexibility conferred by the flexible linkers is better accommodated at the pseudo 3-fold positions of the HBV core shell scaffold (the linkers do not constrain the NadA trimer axis to be precisely aligned to the capsid 3-fold axes). The four chains of the two α-helical spike dimers from the HBc T = 4 core shell crystal structure [30] were rigid body fitted into the core shell map using DockEM [31], as before [29], now implemented in the CCP-EM suite [37], with significant speed-up using the fast local correlation function [31]. We found that the orientation and location of each chain required very little modification from its position in the crystal structure.

To model the core structure more accurately, the residues at positions where the density was not clearly resolved were deleted from the model Protein Data Bank file. This included the unresolved flexible linker and some parts of the connecting HBcS core structure. Helix-3 was truncated to the point the flexible linker ends, at Leu76, and Helix-4a truncated 6 residues after the stabilising base stack between the two subunits, at Arg82. These atomic coordinates were then refined against the 3.4 Å resolution cryoelectron density map using Phenix [38]. Only minor changes in comparison with the native crystal structure were seen [30]: the largest change was a reorientation of helix-4a by 4.5˚. Interestingly, the spike conformation appears stabilized by the base stacking of Trp71 and Tyr88, which could play a role in stabilising the NadA insertion (Fig. 3B).

Electron density for the CFHbp domain appears to be absent. This may be due to steric clashes which could de-stabilize the folded state of the antigen domain: limited space on the interior of the capsid would constrain packing of adjacent CFHbp domain monomers. We cannot rule out, however, that CFHbp retains some tertiary structure and multiple orientations weaken the electron density. In either case, it was recognised that even an unfolded CFHbp domain might retain the ability to elicit the production of antibodies, and was therefore of interest [39].

### 2.3 Assessment of the immunogenicity of chimeric VLPs displaying *Neisseria meningitidis* protein sequences

**Fig. 4:**
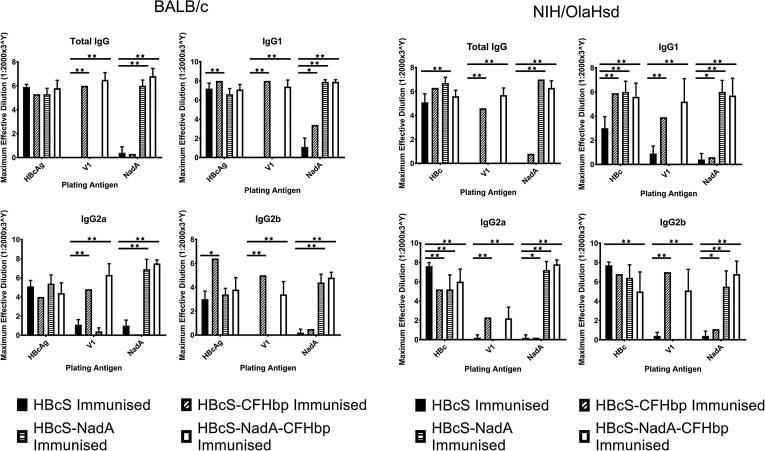
IgG and IgG1 reactivity against native HBc and the HBcS-antigen fusions. ELISA was conducted using plates coated with 100 ng of HBcS, variant 1 FHbp, or NadA followed by serial 3-fold dilution of sera, beginning at 1:2000, from either BALB/c or NIH/OlaHsd mice inoculated with either HBcS or one of the three antigen-containing VLPs (HBcS-CFHbp, HBcS-NadA, or HBcS-NadA-CFHbp). The maximum dilution of serum that surpassed absorption of the non-immunized mouse serum blank was recorded, and the mean average across the immunization group calculated along with 95% confidence intervals. A 2-way ANOVA with multiple comparisons of the mean average reactivity was conducted within each plated antigen group for each immunization group and compared with the negative control HBcS immunization group (excluding self-comparison). * indicates P value < 0.05, ** indicates P value < 0.005.

Mice were immunized with native HBcS and the three HBcS-antigen fusion constructs, using three subcutaneous immunization doses of 0.82 nmol. This is a similar dose, in molar terms, to that used by Giuliani et al. for mouse immunization trials (0.6 and 0.7 nmol for FHbp and NadA respectively), although the comparison is complicated by their use of adjuvant (aluminium hydroxide) and a different mouse strain (CD1) [40]. Both BALB/c and NIH/OlaHsd mouse strains were used in parallel experiments to account for possible variations in responses to antigen. Sera were then analysed by ELISA, using plates coated with immobilized soluble antigen (Fig. 4). From these data, it was possible to distinguish the antibody responses specific for HBcS, variant 1 FHbp or NadA. The plates coated with HBcS served as a positive control. Despite previous reports on strain differences, the general pattern of IgG responses were similar in both BALB/c and NIH/OlaHsd mice. All mice immunized with one of the four constructs developed antibodies against HBcS; fusion of neither CFHbp nor NadA impaired access to epitopes within the HBcS core protein. IgG reactivity against FHbp and NadA was generally consistent with the composition of the immunizing antigen construct. Notably, serum from mice immunized with the dual antigen construct (HBcS-NadA-CFHbp) tended to exhibit the same reactivity against variant 1 FHbp and NadA as the corresponding single variant constructs, both in terms of the titre achieved and subclasses generated. Introduction of a second antigen into the HBcS construct did not therefore seem to impair the immunogenicity of the first antigen.

Serum bactericidal assays (SBAs) were carried out using the mouse sera and baby rabbit complement to determine whether the antibody responses were bactericidal (Table 1). None of the mice immunized with VLPs containing the C-terminal domain of variant 1 FHbp produced bactericidal antibodies, despite the previous successful use of FHbp within vaccine formulations [11], [41]. By contrast, the NadA-containing constructs elicited bactericidal activity greater than the VLP incorporating FHbp alone and the positive control. The *N. meningitidis* strain used (NIBSC 2783) was tested in a whole cell total IgG ELISA using the antisera from BALB/c mice vaccinated with all four HBcS variants (Table S3). The results showed IgG reaction against both CFHbp and NadA, although responses against the former antigen were significantly weaker, which could partially explain the weaker SBA titres.

**Table 1:** SBA titres of sera against *Neisseria meningitidis* from mice inoculated with four different VLP constructs.

	SBA Titre (reciprocal of dilution)^1^	
	BALB/c	NIH/OlaHsd
HBcS	2	2
HBcS-CFHbp	2	2
HBcS-NadA	2048	3072
HBcS-NadA-CFHbp	512	4096
JAR5 mAb	32	3072

1 SBA conducted using baby rabbit complement and sera pooled from ten BALB/c or NIH/OlaHsd mice inoculated with either HBcS or one of the three antigen-containing VLPs (HBcS-CFHbp, HBcS-NadA, or HBcS-NadA-CFHbp) against *Neisseria meningitidis* strain NIBSC 2783. SBA endpoint titre was determined as the last serum dilution able to elicit killing of at least 50% of bacteria, with the median values reported here from two assay repeats. JAR5 mAb (NIBSC catalogue no 13/216) was used as an FHbp positive control and VLP-only sera used as a negative control. Titres are expressed as a reciprocal of the median serum dilution.

Table 2 shows a comparison of the SBA results for HBcS-NadA with two previous reports which used the same allele of recombinant NadA alone [42], [43]. SBA titres for this study are somewhat weaker, by a factor of about 4 for the median NadA titre. Nevertheless, the results show that a meningococcal antigen can be successfully incorporated into a VLP and produce SBA titres which are broadly of the same magnitude as those obtained using antigen alone.

**Table 2:** Comparison of SBA results for NadA antigen.

	This study (HBcS-NadA)	Comanducci et al. 2002	Comanducci et al. 2004
Dose (nmol)	0.82	0.89	0.89
No. of Doses	3	3	3
Adjuvant	None	CFA^1^	Alum or CFA
Mouse Strain	NIH/OlaHsd, BALB/c	CD1	CD1
Bacterial Strains	NIBSC 2783 (92001)	2996, C11, F6124, MC58	2996, 65/96
Complement Source	Baby Rabbit Serum	Baby Rabbit Serum	Baby Rabbit Serum
Median NadA Titre^2^	3072	12,288	12,288
Lowest Reported Titre	1024	4096	2048
Highest Reported Titre	4096	32,768	32,768
Median Control Titre	2	4	80

1 CFA = complete Freund’s adjuvant.

2 All studies use the NadA3 allele. Both Comanducci et al. studies used various bacterial strains for SBA analysis [42], [43]; however, only those SBA titres from strains expressing the NadA3 allele are included here.

## 3 Discussion

Virus-like particles have been the basis of successful, licensed vaccine formulations. However, it is their demonstrated ability to be used as scaffolds for antigen presentation, and to modulate the immune response to a vaccine formulation, that has made them an attractive platform [44], [45], [46], [47]. The consequence of the effect of multiple antigen presentation on immunological responses has been addressed experimentally; it is well established that antigen organization and presentation plays an important part in humoral responses [21], [27]. We have also shown that aggregation of a single chain Fv leads to a change in the Th1/Th2 profile of the induced immune response, relative to monomer [48]. Such work points to the potential in modulating vaccine response through engineering of heterologous antigens into VLPs and similar macromolecular assemblies [49], [50].

The antigens chosen here for inclusion within a VLP, NadA and FHbp, were based on published work underpinning recently approved meningococcal vaccine formulations which identified them as capable of eliciting protective immunity [11], [41]. Several constructs were examined for expression and assembly, incorporating NadA, FHbp and other meningococcal antigens (Table S1). When incorporating a heterologous antigen into a VLP, a key challenge is to ensure that the fusion with the viral structural protein remains competent for assembly of the particle. Preservation of the tertiary structure of the antigen is also an important consideration if the inserted peptide is large enough to form a domain or sub-domain. We found that NadA was the only antigen which could be incorporated into the MIR without compromising VLP expression and assembly. Expression of FHbp as a fusion was only possible using the C-terminal domain incorporated at the HBc C-terminus. Despite this limitation, mice immunized with both the HBc-CFHbp and HBc-NadA-CFHbp constructs elicited IgG antibodies against FHbp (Fig. 4).

Alternative solutions to the problem of antigen incorporation into VLPs have been proposed: for example, independently produced antigens can be attached to the VLP by affinity tag-based coupling [49]. Such methods would require a separate step in the VLP production process, however. Although reports of the use of VLP-antigen fusions as candidate vaccines are common, high resolution investigation of any structural changes to the parent VLP is rare. Peyret *et al.* studied an adapted HBc which had been engineered to favour expression of larger antigen domains: by fusing two consecutive HBc chains, a single antigen- rather than two- is incorporated at the MIR site, increasing the space available thus reducing steric constraints (tandem core) [50]. Folded domains- GFP or a camelid antibody- were inserted and the modified VLPs assembled correctly. Analysis by cryoelectron microscopy showed evidence for density originating from the GFP domain, although it was somewhat diffuse, presumably due to flexibility between the GFP and the core particle.

The cryoelectron microscopy 3D reconstruction of HBc-NadA-CFHbp did not indicate any electron density for the CFHbp domain (Fig. 3). This may reflect disorder of the CFHbp domain relative to the HBc capsid or a failure of the domain to fold. The latter seems more likely: molecular modelling of the CFHbp domain indicates that incorporation of 240 copies onto the interior face of the HBc shell would result in major steric clashes. The electron density for the HBc chain ends abruptly at its C-terminus: even if the CFHbp domain were disordered in orientation, some (weak) density for the chain should be visible, as is the case for NadA. Failure to adopt a folded conformation could have eliminated crucial conformational epitopes involved in promoting antibody avidity, which normally assist in complement-mediated bacteriolysis by the classical pathway [51] and partly explains the weak SBA response against the CFHbp domain. In light of these results, we propose that the ability of HBcS-FHbp-NadA to induce a bactericidal response is driven by the NadA, rather than the FHbp component.

The detection of IgG antibodies against FHbp also raises the question how, if the domain is located on the interior of the particle, it stimulates an antibody response. Dishlers *et al.* showed that removal of the Arg-rich peptide from the HBc C-terminus and substitution of a Gly linker enabled the surface exposure of an inserted peptide epitope [33]. The linkers used in the constructs reported here are also Gly-based (Fig. 1). It is possible that, in a minority of cases, some CFHbp domains are surface-exposed. Alternatively, a few incorrectly assembled particles could provide sufficient CFHbp domains to trigger a response. The finding that our disordered CFHbp domains elicited production of antibodies offers some encouragement that a VLP construct containing either a full-length or domain-length FHbp insert displayed in its native orientation may achieve better SBA titers. Domain insertion within the MIR, with suitably designed ‘linker regions, could be used in combination with modelling and screening using antibodies specific for FHbp in its folded state.

We anticipated that it would be challenging to introduce NadA into the HBc VLP without compromising its assembly. NadA forms an elongated trimeric structure with a small head domain, stabilized through a coiled coil stem [18]. Protective epitopes are thought to be in the head domain [18]. The MIR sits at the tip of two α-helices within the HBc chain, which associate with their counterparts from a second HBc monomer on a 2-fold symmetry axis [30]. Therefore insertion of NadA into the MIR would generate a symmetry mismatch (Fig. 1C&D). Surprisingly, the HBc assembly accommodates this modification, although the stability, as estimated from the Thermofluor assay (Fig. 2C), is compromised. Nevertheless, the HBc-NadA-CFHbp construct is about 3.7-fold higher in mass than the parent HBc and is evidence of the robust nature of the capsid assembly. The detection of electron density for NadA on the 3-fold symmetry axes (Fig. 3) suggests that NadA is partially folded - at least sufficiently to allow formation of a trimer. This proposal is supported by evidence for a greater helical content (Fig. 2D). Retention of conformational epitopes in NadA probably explains the higher SBA titres obtained using the NadA-containing constructs (Table 1).

We examined IgG antibody responses to vaccination with the HBc constructs in both BALB/c and NIH/OlaHsd mouse strains (Fig. 4). Previous investigators have reported different immune responses, depending on the mouse strain used; for example, the bias toward Th2 responses in BALB/c mice is well documented [52]. However, our data indicate little difference in IgG responses between the mouse strains.

Based on the stark difference in the ability to induce the production of bactericidal antibodies between VLP constructs incorporating CFHbp, NadA, or both antigens, we hypothesize that the state of folding of the antigen can be crucial in inducing the desired immune response. Furthermore, despite minor differences between our study and previous work, the data show that it is possible to obtain SBA titres for HBcS-NadA which are of a similar magnitude to those obtained with the separate protein. It is possible that further work to optimize, for example, CFHbp or NadA structural integrity within the VLP could remediate or improve the SBA titres respectively.

This work has set the basis for future investigations using HBc VLPs as a platform for the incorporation of *Neisseria* antigen sequences incorporating complete, folded domains. In particular, optimising the orientation of the NadA sequence in the HBcS-NadA could be useful in identifying constructs that are more likely to yield VLPs displaying antigens in a native-like folded state, and hence are more likely to induce a bactericidal antibody response.

## 4 Methods

### 4.1 Expression and purification of VLPs

Details of the design of VLP constructs can be found in Table S1. The relevant sequence for each VLP construct was cloned into the pET-17b expression plasmid and transformed into BL21 ClearColi *E. coli* (Lucigen) for expression. Transformed cells were cultured in 50 ml LB Broth medium, 100 µg/ml ampicillin, prior to transfer to large scale shaker flask cultures(450 ml 2 × YT medium, 100 µg/ml ampicillin) at 37 °C, 200 rpm until cell density reached OD_600_ = 0.8. At that point, protein expression was induced by addition of 0.1 mM IPTG (final concentration) and the cultures were grown overnight at 16 °C, 200 rpm. Cells were harvested by centrifugation at 11,000*g* for 20 mins at 4 °C and resuspended at a ratio of 4 ml/g cells in Strep-Wash buffer (100 mM Tris, 150 mM NaCl, 1 mM EDTA, pH 8.0). Protease inhibitor tablets were added along with DNase I 5µg/ml to improve protein recovery and reduce chromatography resin fouling. The cell suspension was then lysed by sonication on ice using the Bandelin Sonopuls HD3100 with UW3200 converter at 35% amplitude for 10 mins of 5 s pulses with 10 s rests. Cellular debris was removed by centrifugation at 38,000*g* for 40 mins at 4 °C, and the supernatant was filtered through both 0.45 µm and 0.2 µm PES filters before Strep-Tag affinity chromatography with 5 ml fraction collection. Sample was applied to the 5 ml StrepTrap HP column, pre-equilibrated with 25 ml Strep-Wash bufferat a flow rate of 1 ml/min. This was followed by 50 ml Strep-Wash at 2 ml/min and subsequent elution with 25 ml Strep-Wash buffer plus 2.5 mM desthiobiotin at 1 ml/min. Fractions identified as containing target protein by SDS-PAGE were concentrated by diafiltration to a volume of 1 ml before filtration through a 0.22 µm PES filter. Further purification was conducted by size exclusion chromatography using a Superose6 column (GEHealthcare), in 1 × PBS (pH 8.0) buffer at a flow rate of 0.5 ml/min. Fractions identified as containing target protein by SDS-PAGE were pooled and the protein concentration determined from absorption at 280 nm. Samples were stored at −80 °C. Methods for the preparation of recombinant FHbp and NadA, the Thermofluor assay and all electron microscopy method details are in Supplementary Materials.

### 4.2 Circular dichroism

This assay was conducted using the Spectra Manager software with the Jasco J-810 Spectropolarimeter. Protein samples and corresponding buffer blanks were centrifuged at 13,000*g* for 1 min to remove any particulate matter, before loading 180 µl of sample into a 0.5 mm UV-quartz cell cuvette. A run protocol was then executed using the following parameters: absorption spectrum from 240 nm to 190 nm; 1 nm read intervals; bandwidth = 1 nm; response = 0.5 s; accumulation = 10; cell length = 0.05 cm. The buffer blank spectra were then subtracted from the protein containing spectra and the results analysed using DichroWeb [53].

### 4.3 Animal experiments

Female BALB/c and NIH/OlaHsd-Swiss strain mice, 6–8 weeks old (Envigo, UK) were used in the study. 10 mice of each strain per construct were immunized by subcutaneous (s.c.) injection with the VLP construct in a volume of 200 µl (0.82 nmoles) diluted appropriately in PBS on days 0, 21, 35. Blood was collected on day 49 by cardiac puncture under terminal anaesthesia. Serum was separated by centrifugation at 9600 × g for 10 min and stored at −20 °C until use. All procedures were conducted in accordance with UK Home Office regulations under licence number 80/2634 and were approved by the National Institute for Biological Standards and Control ethics committee.

### 4.4 Serum ELISA

Serum ELISA was conducted in Nunc Maxisorp flat-bottom 96-well plates, with one of the following anti-mouse secondary HRP-conjugated antibodies produced in goat: Total IgG (Sigma-Aldrich), IgG1 (Southern Biotech), IgG2a (Invitrogen), IgG2b (Invitrogen). All wells were incubated overnight at 4 °C with 100 µl coating antigen (HBcS, variant 1 FHbp, or NadA) at a concentration of 1 µg/ml (plating antigen details in Supplementary Data Table S2). The plate was then inverted on absorbent tissue and allowed to completely drain, tapping out forcefully if necessary before washing three times (1 × PBS, 0.02% Tween-20) to remove residual coating antigen. The coated plates were blocked for 1hr at 20 °C with 200 µl Blocking Buffer (1 × PBS, 5% Foetal Bovine Serum), which was then inverted on absorbent tissue and allowed to completely drain. Rows B-H were filled with 100 µl Blocking Buffer, and 150 µl of sera. Controls (negative: RmpM-immunized mouse serum, positive = pooled sera) at 1:2000 dilution were then aliquoted into wells on row A. Then, beginning with row A, each row was diluted 3-fold into the succeeding row by addition of 50 µl from one row into the next. After these serial dilutions, 50 µl from row H was then discarded to ensure all wells contained 100 µl, and the plates were incubated for 2hrs at 20 °C. The plates were then washed three times with Wash Buffer (as above), 100 µl of secondary antibody at 1:1000 dilution was added and wells incubated for 2hrs at 20 °C. The plates washed three times with Wash Buffer, 100 µl TMBlue stabilized chromagen (Thermo Fisher Scientific)added, incubated for 10mins at 20 °C before termination of the reaction by addition of 100 µl 1 M HCl. Absorption of the reaction wells was then measured at 450 nm. Serum activity was defined as the lowest serum dilution with an absorption reading greater than the corresponding well in the negative control lane. Serum dilution was then converted to a 0–8 scale, with 0 corresponding to activity lower than 1:2000 dilution, and 8 corresponding to activity greater than 1:4,374,000 dilution.

### 4.5 Serum bactericidal assay (SBA)

A modified protocol was used to measure bactericidal activity of mice sera [54]. Following a screen of 6 commonly used *N. meningtidis* strains for FHbp and NadA expression using anti- FHbp and anti-NadA sera, NIBSC strain 2783 was selected as optimal for expression of both proteins and hence used as the target strain for SBA. Genome sequence data for this strain, available from the PubMLST Neisseria spp. Database, confirms 100% sequence identity with the FHbp and NadA sequences cloned for the VLP constructs. *N. meningitidis* strain NIBSC 2783 was grown overnight on Muller Hinton Blood Agar (MHBA) with 5% horse blood (Oxoid, Thermo Fisher Scientific, TCS) followed by replating onto a fresh MHBA agar. After incubating at 37 °C, 5% CO_2_ for 4 h, bacteria were resuspended in bactericidal buffer (Gey's Balanced Salt Solution with 0.5% BSA, Sigma-Aldrich) to an OD_600nm_ of ~ 0.2. The suspension was further diluted 1 in 2500 to obtain a cell concentration of approximately 5 × 10^4^ cells/ml. Equal volumes (10 µl) of the diluted cells and baby rabbit complement (Pel-Freeze Biologicals) were added to 20 µl test sera, serially diluted two-fold in bactericidal buffer in 96-well U-bottomed microtiter plates (Greiner, Frickenhausen, Germany). The reaction mixture was gently mixed by tapping and incubated at 37 °C, 5% CO_2_ for 1 h. Using a multichannel pipette, 10 µl from each well was removed and allowed to flow in lanes from one end of the square MHBA plate to the other end (Tilt method) and incubated overnight at 37 °C, 5% CO_2_. The colony forming units (CFU) in different serum dilutions were counted and compared with CFUs observed for the control wells without test serum or with heat-inactivated rabbit complement. The reciprocal of the serum dilution that resulted in 50% killing relative to no serum control was assigned as SBA titre for each sample.

## 5 Data availability

The electron microscopy maps have been deposited in the EMDB with accession number EMD-10316. The modelled coordinates are deposited in the Protein Data Bank with accession number 6TIK.

## CRediT authorship contribution statement

**Sebastian Aston-Deaville:** Conceptualization, Investigation, Formal analysis, Visualization, Writing - original draft. **Emil Carlsson:** Investigation, Formal analysis, Writing - review & editing. **Muhammad Saleem:** Investigation, Formal analysis, Writing - review & editing. **Angela Thistlethwaite:** Investigation. **Hannah Chan:** Investigation, Formal analysis, Writing - review & editing. **Sunil Maharjan:** Investigation, Formal analysis, Writing - review & editing. **Alessandra Facchetti:** Investigation, Formal analysis, Writing - review & editing. **Ian M. Feavers:** Conceptualization, Resources, Project administration, Writing - review & editing. **C. Alistair Siebert:** Investigation, Formal analysis. **Richard F. Collins:** Investigation, Formal analysis, Writing - review & editing. **Alan Roseman:** Investigation, Formal analysis, Visualization, Writing - review & editing. **Jeremy P. Derrick:** Conceptualization, Data curation, Formal analysis, Funding acquisition, Project administration, Resources, Supervision, Visualization, Writing - review & editing.

## Declaration of Competing Interest

The authors declare that they have no known competing financial interests or personal relationships that could have appeared to influence the work reported in this paper.
