# Supplementary material for: An assessment of the use of Hepatitis B Virus core protein virus-like particles to display heterologous antigens from Neisseria meningitidis
Source: Vaccine. 2020 Apr 3;38(16):3201–9. doi: 10.1016/j.vaccine.2020.03.001 (PMC7113836; doi:10.1016/j.vaccine.2020.03.001)
Supplement: Supplementary data 1 [file mmc1.docx]

**Supplementary Methods**

Expression and Purification of FHbp and NadA

Recombinant antigens FHbp and NadA were expressed and purified as follows: the relevant sequence for each construct was cloned into the pET-22b expression plasmid and transformed into T7 E.coli (New England Biolabs) for expression. Transformed cells were cultured in 50ml LB Broth medium, 100µg/ml ampicillin, prior to transfer to large scale shaker flask cultures(450ml 2×YT medium, 100µg/ml ampicillin) at 37°C, 200rpm until cell density reached OD600 = 0.8. At that point, protein expression was induced by addition of 0.1mM IPTG (final concentration) and the cultures were grown overnight at 16°C, 200rpm. Cells were harvested by centrifugation at 11,000g for 20 mins at 4˚C and resuspended at a ratio of 4ml/g cells in HisTag-Wash buffer (50mM Tris, 100mM NaCl, pH 8.0). Protease inhibitor tablets were added along with DNase I 5µg/ml to improve protein recovery and reduce chromatography resin fouling. The cell suspension was then lysed by sonication on ice using the Bandelin Sonopuls HD3100 with UW3200 converter at 35% amplitude for 10 mins of 5s pulses with 10s rests. Cellular debris was removed by centrifugation at 38,000g for 40 mins at 4˚C, and the supernatant was filtered through both 0.45µm and 0.2µm PES filters before His-Tag affinity chromatography with 5ml fraction collection. Sample was incubated with 1ml Ni-NTA resin per 40ml lysate, pre-equilibrated with 25ml HisTag-Wash buffer, for 1 hour at 4˚C on a rotary platform. This was followed by sequential 10ml chromatography steps employing a 10 mins incubation and collection process at the following concentrations of imidazole: 0mM, 20mM, 40mM, 2 x 200mM, and 500mM. Fractions identified as containing target protein by SDS-PAGE were dialysed overnight 4˚C against a volume of HisTag-Wash buffer 30-50 X greater, before filtration through a 0.22µm PES filter. Further purification was conducted by size exclusion chromatography using a Superdex200 column (GEHealthcare), in 1×PBS (pH 8.0) buffer at a flow rate of 0.5ml/min. Fractions identified as containing target protein by SDS-PAGE were pooled and the protein concentration determined from absorption at 280nm. Samples were stored at -80˚C.

## Thermofluor Assay

This assay was conducted using the 96-well plate compatible Step One Plus qPCR machine and software (Life Technologies). Reaction aliquots, performed in triplicate, were composed as follows: fluorescent SYPRO orange dye at 5× concentration, protein sample (excluding the blank) at 25 µg/ml and1×PBS (pH 8.0) 20µl total volume. Heating conditions were as follows: equilibration of the reaction plate at 25˚C for 15 s, followed by a linear temperature ramp rate of 1.32 °C per minute from 25˚C to 99.9°C and, finally, equilibration of the reaction plate at 99.9˚C for 15 s. Fluorescence measurements were recorded by the machine calibrated for use with the ROX dye (which has major overlap of emission spectrum with SYPRO orange), and the protein sample data was automatically normalized to the blank.

## Electron Microscopy

For initial evaluation by negative staining, samples (1mg/ml) were adhered to carbon-coated 400 mesh copper grids and then stained with 2% (w/v) uranyl acetate. TEM images were acquired using a FEI Tecnai 12 BioTwin and a Gatan Orius camera. Samples prepared for cryoelectron microscopy were adhered to glow discharged QUANTIFOIL grids (R 2/2 400 Mesh Copper) by incubation of a 3 μl droplet on the grid for 30 s. The grids were then continuously blotted for 4–5 s in a 90% humidity chamber, before plunge-freezing into liquid ethane. Images were acquired using the FEI Tecnai G2 Polara operating at 300 kV on a 4K Gatan Ultrascan Charge Coupled Device (CCD) in low-dose mode (FEI) for screening purposes. The same grids were then used for data collection in a Titan KRIOS – data were collected in low dose mode at 300 kV using a FALCON III Direct Detection Device (DDD). 40-image-stacks providing a total dose of ~80 electrons were collected per image and dose weighted using Motioncor2[[1](#_ENREF_1)]. Initial image processing was performed using EMAN2 [[2](#_ENREF_2)], applied to contrast transfer function (CTF) corrected data, using strategies previously described [[3](#_ENREF_3), [4](#_ENREF_4)]. Initially 44,000 particles were selected using swarm parameters in E2Boxer, followed by manual refinement wherein overlapping and deformed particles were discarded. Data were then CTF-corrected and, following class averaging, classes of particles forming complete, non-deformed icosahedral shells were selected visually. Preliminary models were generated from 10119 selected particles, subject to twelve rounds of iterative refinement, yielding a density map. Resolution was estimated, as has been done previously, from the Fourier shell correlation [[5](#_ENREF_5)]. The resultant CTF corrected particle stack from EMAN, with associated metadata, was then exported to Cryosparc[[6](#_ENREF_6)]. In Cryosparc one round of 2D classification was done, and particles from a few low quality classes were excluded. An initial run of heterogeneous multimodel refinement (with 3 models) was performed for further cleaning of the particle set. Then the 95% of particles assigned to the best resolution structure were refined to their model in homogeneous refinement, yielding a map with an overall resolution of 6Å, from 8,598 particles. One more round of 3D refinement was performed, using a mask to define the VLP core region, which yielded a 3.4 Å resolution map. All refinement was carried out applying icosahedral symmetry.

Model building

The 4 protein chains from the original T=4 HBV core shell structure solved by X-ray crystallography [[7](#_ENREF_7)], Protein Data Bank entry ID 1QGT, representing two independently resolved dimeric spikes, were docked into the map using DockEM [12], and visualised in UCSF Chimera. Part of the chains near the tips of the spikes did not fit well to the map. This region of the map, corresponding to the major immune dominant region where the inserted NadA epitope was placed, was poorly resolved. Therefore the model region according to the native sequence between, but not including, Leu76 to Arg82 was deleted from all 4 chains. This model was then refined against the map with the phenix.real_space_refine program, using global minimisation, simulated annealing, B-factor refinement, and without non-crystallographic symmetry (NCS) restraints [[8](#_ENREF_8)]. Default parameters were used for other constraints. The resulting model was re-refined once using the local_grid_search option, with all other parameters left unchanged.

**Supplementary Tables**

| **Construct Name** | **Purification Tag** | **Region of Antigen Insertion** | **Confirmed Expression in *E.coli*** | **Successful Purification by Strep-Tag Affinity** | **Inferred Successful Formation of VLP** |
| --- | --- | --- | --- | --- | --- |
| HBcS | Strep-Tag | - | ✔ | ✔ | ✔ |
| HBcS-CFHbp (V1) | Strep-Tag | C-terminus | ✔ | ✔ | ✔ |
| HBcS-NadA | Strep-Tag | MIR | ✔ | ✔ | ✔ |
| HBcS-NadA-CFHbp | Strep-Tag | MIR and C-terminus | ✔ | ✔ | ✔ |
| HBcS-CFHbp(V3) | Strep-Tag | C-terminus | ✖ | ✖ | ✖ |
| HBcS-NHBA[[9](#_ENREF_9)] | Strep-Tag | MIR | ✔ | ✖ | ✖ |
| HBcS-GNA1162[[10](#_ENREF_10)] | Strep-Tag | MIR | ✔ | ✖ | ✖ |
| HBcS-NMB0928[[11](#_ENREF_11)] | Strep-Tag | MIR | ✔ | ✖ | ✖ |
| HBcS-Mosaic-Cst\|V3 | I. Strep-Tag II. None | II. C-terminus | ✔ | ✔ | ✔ |
| HBcS-Mosaic-V1\|V3 | I. Strep-Tag II. FLAG-Tag | C-terminus | ✔ | ✔ | ✔ |
| HBcS-FHbp (V1) | Strep-Tag | C-terminus | ✔ | ✔ | ✖ |
| HBcS-NFHbp (V1) | Strep-Tag | C-terminus | ✔ | ✔ | ✖ |

Table S1. Summary of the VLP constructs trialled. Structural features that were expected to influence purification and details of the success experienced during expression and purification of each construct were noted. Successful formation of the VLP was inferred from identification of a peak in the void volume of size exclusion chromatography that was verified as containing VLP protein by SDS-PAGE (for details see Methods). Construct name abbreviations defined as follows: Cst = HBcS with no addition, V1 = HBcS incorporating FHbp full length, CFHbp and NFHbp sequence from Variant 1 subtype FHbp, V3 = HBcS incorporating CFHbp sequence from Variant 3 subtype FHbp.

| **HBcS** |
| --- |
| MDIDPYKEFGATVELLSFLPSDFFPSVRDLLDTASALYREALESPEHCSPHHTALRQAILCWGELMTLATWVGNNLEDASRDLVVNYVNTNMGLKIRQLLWFHISCLTFGRETVLEYLVSFGVWIRTPPAYRPPNAPILSTLPETTVVGSWSHPQFEK |
| **HBcS-CFHbp**  fHbp sequence (Q9JXV4_NEIMB) from MC58 aa 203-320 |
| MDIDPYKEFGATVELLSFLPSDFFPSVRDLLDTASALYREALESPEHCSPHHTALRQAILCWGELMTLATWVGNNLEDASRDLVVNYVNTNMGLKIRQLLWFHISCLTFGRETVLEYLVSFGVWIRTPPAYRPPNAPILSTLPETTVVGSGGGHTSFDKLPEGGRATYRGTAFGSDDAGGKLTYTIDFAAKQGNGKIEHLKSPELNVDLAAADIKPDGKRHAVISGSVLYNQAEKGSYSLGIFGGKAQEVAGSAEVKTVNGIRHIGLAAKQGGGWSHPQFEK |
| **HBcS-NadA** NadA sequence (Q9JXK7_NEIMB) from MC58 aa26-309 |
| MDIDPYKEFGATVELLSFLPSDFFPSVRDLLDTASALYREALESPEHCSPHHTALRQAILCWGELMTLATWVGNNLEDGGGGSGGGGSATSDDDVKKAATVAIVAAYNNGQEINGFKAGETIYDIGEDGTITQKDATAADVEADDFKGLGLKKVVTNLTKTVNENKQNVDAKVKAAESEIEKLTTKLADTDAALADTDAALDETTNALNKLGENITTFAEETKTNIVKIDEKLEAVADTVDKHAEAFNDIADSLDETNTKADEAVKTANEAKQTAEETKQNVDAKVKAAETAAGKAEAAAGTANTAADKAEAVAAKVTDIKADIATNKADIAKNSARIDSLDKNVANLRKETRQGLAEQAALSGLFQPYNVGEFGGGGSGGGGSRDLVVNYVNTNMGLKIRQLLWFHISCLTFGRETVLEYLVSFGVWIRTPPAYRPPNAPILSTLPETTVVGSGGGTGKLGGGWSHPQFEK |
| **HBcS-NadA-CFHbp**  NadA sequence (Q9JXK7_NEIMB) from MC58 aa26-309  fHbp sequence (Q9JXV4_NEIMB) from MC58 aa 203-320 |
| MDIDPYKEFGATVELLSFLPSDFFPSVRDLLDTASALYREALESPEHCSPHHTALRQAILCWGELMTLATWVGNNLEDGGGGSGGGGSATSDDDVKKAATVAIVAAYNNGQEINGFKAGETIYDIGEDGTITQKDATAADVEADDFKGLGLKKVVTNLTKTVNENKQNVDAKVKAAESEIEKLTTKLADTDAALADTDAALDETTNALNKLGENITTFAEETKTNIVKIDEKLEAVADTVDKHAEAFNDIADSLDETNTKADEAVKTANEAKQTAEETKQNVDAKVKAAETAAGKAEAAAGTANTAADKAEAVAAKVTDIKADIATNKADIAKNSARIDSLDKNVANLRKETRQGLAEQAALSGLFQPYNVGEFGGGGSGGGGSRDLVVNYVNTNMGLKIRQLLWFHISCLTFGRETVLEYLVSFGVWIRTPPAYRPPNAPILSTLPETTVVGSGGGTHTSFDKLPEGGRATYRGTAFGSDDAGGKLTYTIDFAAKQGNGKIEHLKSPELNVDLAAADIKPDGKRHAVISGSVLYNQAEKGSYSLGIFGGKAQEVAGSAEVKTVNGIRHIGLAAKQKLGGGWSHPQFEK |
| **Variant 1 FHbp**  fHbp sequence (Q9JXV4_NEIMB) from MC58 residues 77-320 |
| MIGAGLADALTAPLDHKDKGLQSLTLDQSVRKNEKLKLAAQGAEKTYGNGDSLNTGKLKNDKVSRFDFIRQIEVDGQLITLESGEFQVYKQSHSALTAFQTEQIQDSEHSGKMETVAKRQFRIGDIAGEHTSFDKLPEGGRATYRGTAFGSDDAGGKLTYTIDFAAKQGNGKIEHLKSPELNVDLAAADIKPDGKRHAVISGSVLYNQAEKGSYSLGIFGGKAQEVAGSAEVKTVNGIRHIGLAAKQLEHHHHHH |
| **NadA**  NadA sequence (Q9JXK7_NEIMB) from MC58 residue 26 |
| ATSDDDVKKAATVAIVAAYNNGQEINGFKAGETIYDIGEDGTITQKDATAADVEADDFKGLGLKKVVTNLTKTVNENKQNVDAKVKAAESEIEKLTTKLADTDAALADTDAALDETTNALNKLGENITTFAEETKTNIVKIDEKLEAVADTVDKHAEAFNDIADSLDETNTKADEAVKTANEAKQTAEETKQNVDAKVKAAETAAGKAEAAAGTANTAADKAEAVAAKVTDIKADIATNKADIAKNSARIDSLDKNVANLRKETRQGLAEQAALSGLFQPYNVGR |

Table S2. Sequences of VLP and antigen constructs.

|  | **HBcS** | **HBcS-CFHbp** | **HBcS-NadA** | **HBcS-NadA-CFHbp** |
| --- | --- | --- | --- | --- |
| **1/500** | 0.107 | 0.881 | 3.042 | 3.067 |
| **1/1000** | 0.050 | 0.471 | 2.903 | 3.154 |
| **1/2000** | 0.022 | 0.248 | 2.427 | 2.660 |
| **Blank** | -0.005 | 0.022 | -0.009 | -0.007 |

Table S3. Total IgG reactivity against N. meningitidis NIBSC 2783. Absorbance data at 450nm were obtained from whole cell ELISA conducted on NIBSC 2783 immobilised on the plate. Total IgG was detected using sera from BALB/c mice (see Figure 4).

**Supplementary Figures**


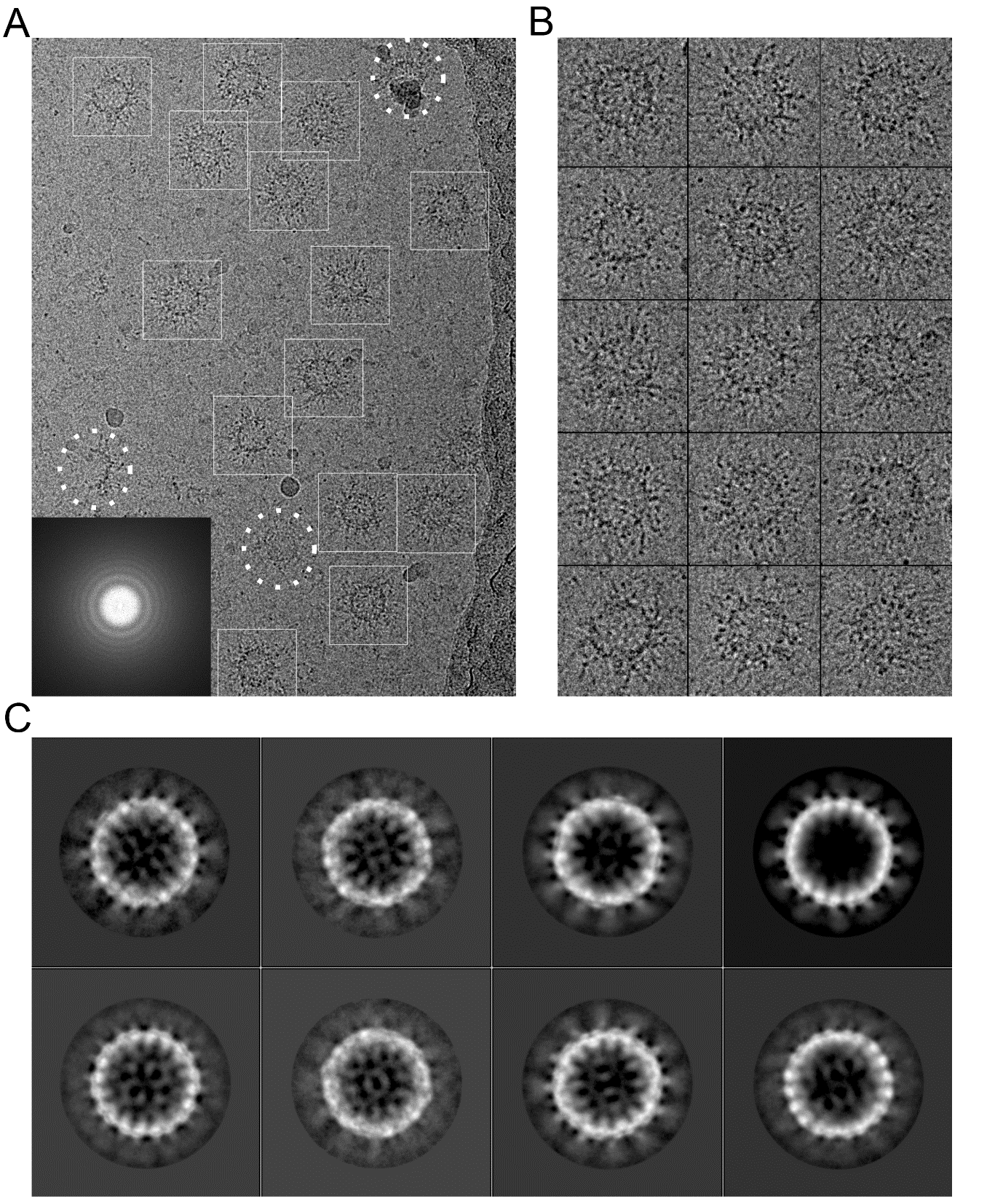


**Figure S1 Cryoelectron microscopy structure determination of HBcS-NadA-CFHbp.**

(A) Field of view of example VLP particles embedded in a layer of vitreous ice. Particles selected for analysis are shown in 448 pixel square boxes (627 Å^2^). Partial folds, shell fragments and contaminated particles are shown in dotted circles and were discarded. The inset box shows a power spectrum of data with sharp Thon rings extending past 4 Å resolution. (B) Individual example particles from (A) selected for analysis. Density for the NadA antigen on the surface can be seen as filamentous structures protruding from the shell surface. Box size is the same as (A). (C) Reference-free class averages from the initially selected data calculated in RELION. Box size is the same as (A), with a 460 Å diameter mask applied to the initial refinement. The icosahedral organisation of the shell core is readily apparent and the NadA can be seem emerging from the shell surface as a cone-shaped funnel of density; as the density moves further from the surface the blur increases, indicating flexibility.

**Figure S2 Resolution plot.** The resolution of the core shell part of the density map is 3.4 Å, using the 0.143 Fourier shell correlation (FSC) criterion.


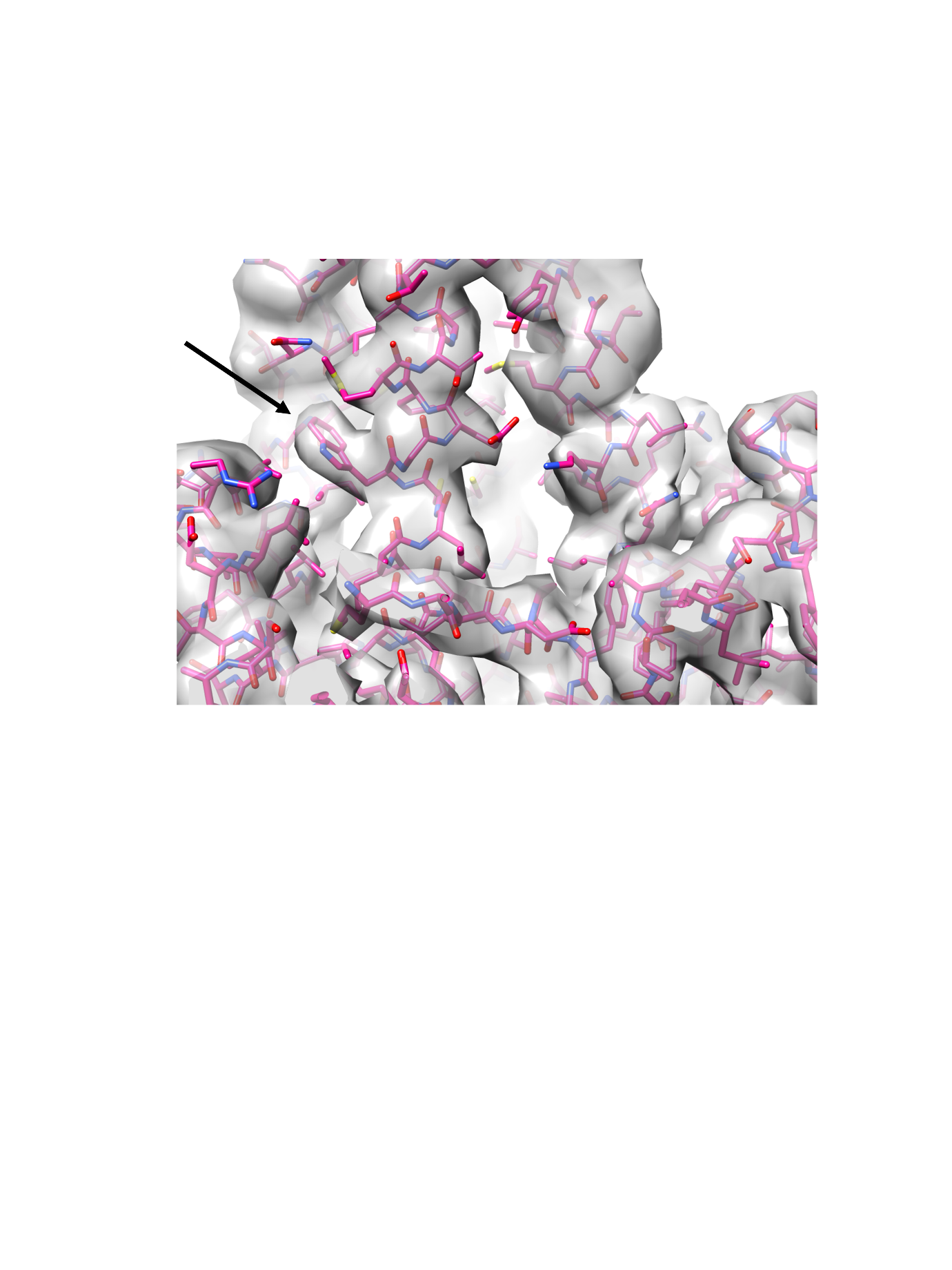


**Figure S3 Detail of the modelled structure of the core protein.** Side chains, such as the tryptophan indicated by arrow, are clearly resolved.

**References**

[1] Peng HC, Zhou Z, Meijering E, Zhao T, Ascoli GA, Hawrylycz M. Automatic tracing of ultra-volumes of neuronal images. Nature Methods. 2017;14:332-3.

[2] Tang G, Peng L, Baldwin PR, Mann DS, Jiang W, Rees I, et al. EMAN2: An extensible image processing suite for electron microscopy. J Struct Biol. 2007;157:38-46.

[3] Roseman AM, Borschukova O, Berriman JA, Wynne SA, Pumpens P, Crowther RA. Structures of Hepatitis B Virus Cores Presenting a Model Epitope and Their Complexes with Antibodies. Journal of Molecular Biology. 2012;423:63-78.

[4] Collins RF, Hassan D, Karuppiah V, Thistlethwaite A, Derrick JP. Structure and mechanism of the PilF DNA transformation ATPase from Thermus thermophilus. Biochem J. 2013;450:417-25.

[5] Collins RF, Frye SA, Kitmitto A, Ford RC, Tonjum T, Derrick JP. Structure of the Neisseria meningitidis outer membrane PilQ secretin complex at 12 angstrom resolution. J Biol Chem. 2004;279:39750-6.

[6] Punjani A, Rubinstein JL, Fleet DJ, Brubaker MA. cryoSPARC: algorithms for rapid unsupervised cryo-EM structure determination. Nature Methods. 2017;14:290.

[7] Wynne SA, Crowther RA, Leslie AGW. The crystal structure of the human hepatitis B virus capsid. Mol Cell. 1999;3:771-80.

[8] Afonine PV, Poon BK, Read RJ, Sobolev OV, Terwilliger TC, Urzhumtsev A, et al. Real-space refinement in PHENIX for cryo-EM and crystallography. Acta Crystallographica Section D-Structural Biology. 2018;74:531-44.

[9] Serruto D, Spadafina T, Ciucchi L, Lewis LA, Ram S, Tontini M, et al. Neisseria meningitidis GNA2132, a heparin-binding protein that induces protective immunity in humans. Proc Natl Acad Sci U S A. 2010;107:3770-5.

[10] Cai XY, Lu J, Wu ZH, Yang CT, Xu HL, Lin ZJ, et al. Structure of Neisseria meningitidis lipoprotein GNA1162. Acta Crystallographica Section F-Structural Biology Communications. 2013;69:362-8.

[11] Delgado M, Yero D, Niebla O, Gonzalez S, Climent Y, Perez Y, et al. Lipoprotein NMB0928 from Neisseria meningitidis serogroup B as a novel vaccine candidate. Vaccine. 2007;25:8420-31.

[12] Roseman, A.M. (2000). Docking structures of domains into maps from cryo-electron microscopy using local correlation. Acta Cryst. D56, 1332-1340.
